# Development of a minimization instrument for allocation of a hospital-level performance improvement intervention to reduce waiting times in Ontario emergency departments

**DOI:** 10.1186/1748-5908-4-32

**Published:** 2009-06-08

**Authors:** Chad Andrew Leaver, Astrid Guttmann, Merrick Zwarenstein, Brian H Rowe, Geoff Anderson, Therese Stukel, Brian Golden, Robert Bell, Dante Morra, Howard Abrams, Michael J Schull

**Affiliations:** 1 Institute for Clinical Evaluative Sciences, 2075 Bayview Ave, Toronto, Canada; 2 Department of Paediatrics, University of Toronto, Toronto, Canada; 3 Department of Health Policy, Management and Evaluation, University of Toronto, Toronto, Canada; 4 Centre for Health Services Sciences, Sunnybrook Health Sciences Centre, 2075 Bayview Ave, Toronto, Canada; 5 Department of Emergency Medicine and School of Public Health, University of Alberta, Edmonton, Canada; 6 Rotman School of Management, University of Toronto, Toronto, Canada; 7 University Health Network, 90 Elizabeth St, Toronto, Canada; 8 Department of Medicine, University of Toronto, Toronto, Canada; 9 Mount Sinai Hospital, 600 University Ave, Toronto, Canada; 10 Clinical Epidemiology Unit, Sunnybrook Health Sciences Centre, 2075 Bayview Ave, Toronto, Canada

## Abstract

**Background:**

Rigorous evaluation of an intervention requires that its allocation be unbiased with respect to confounders; this is especially difficult in complex, system-wide healthcare interventions. We developed a short survey instrument to identify factors for a minimization algorithm for the allocation of a hospital-level intervention to reduce emergency department (ED) waiting times in Ontario, Canada.

**Methods:**

Potential confounders influencing the intervention's success were identified by literature review, and grouped by healthcare setting specific change stages. An international multi-disciplinary (clinical, administrative, decision maker, management) panel evaluated these factors in a two-stage modified-delphi and nominal group process based on four domains: change readiness, evidence base, face validity, and clarity of definition.

**Results:**

An original set of 33 factors were identified from the literature. The panel reduced the list to 12 in the first round survey. In the second survey, experts scored each factor according to the four domains; summary scores and consensus discussion resulted in the final selection and measurement of four hospital-level factors to be used in the minimization algorithm: improved patient flow as a hospital's leadership priority; physicians' receptiveness to organizational change; efficiency of bed management; and physician incentives supporting the change goal.

**Conclusion:**

We developed a simple tool designed to gather data from senior hospital administrators on factors likely to affect the success of a hospital patient flow improvement intervention. A minimization algorithm will ensure balanced allocation of the intervention with respect to these factors in study hospitals.

## Introduction

### Balancing potential confounders in evaluation of hospital-level interventions

Rigorous evaluation of an intervention requires that its allocation be unbiased with respect to confounders. Randomization provides a mechanism for ensuring that intervention and control groups are balanced in terms of both measured and unmeasured confounders. However, if the sample size for the intervention is small there still may be substantial imbalance in the distribution of key confounders due to random error. One way to help circumvent this problem is to stratify or match on key characteristics before randomization. In order for this to work, a small but inclusive set of key potential confounders must be identified.

This paper describes a modified-delphi and nominal group process that resulted in the development of a short survey instrument that defines potential confounding factors likely to influence the success of a hospital-level intervention to improve patient flow in order to reduce emergency department length-of-stay. The purpose of the instrument is to guide the dynamic randomization of participating hospitals to the intervention, using the method of minimization. Dynamic randomization, enabled by the method of minimization, is a widely accepted randomization approach in clinical and multi-institutional trials [1-5]. The minimization method begins with the determination of a small number of factors known or believed to confound the effect of the intervention. The method assigns subjects to a balanced allocation sequence or to treatment groups with respect to marginal frequencies between these selected covariates. This is achieved by an algorithm that allocates the intervention to each subject, in our case, a hospital, that volunteers and is eligible to receive the intervention [6-8].

### Overview of the intervention being evaluated

Every year in Canada more than 12 million emergency department (ED) visits are made,[9] and about a quarter of Canadians visit an ED for themselves or a close family member [10]. Recently, prolonged waiting times in EDs have been the subject of much debate in Canada and elsewhere, and several jurisdictions have launched interventions to reduce them. In 2008, the Ontario Ministry of Health (MOH) announced a provincial ED 'wait times strategy' designed to improve ED patient wait times, patient flow and patient satisfaction. The strategy includes an 'Emergency Department Process Improvement Program' (ED-PIP), a hospital-level intervention intended to improve hospital processes for admitted ED patients in order to improve access to in-patient beds and reduce ED waiting times [11-15].

The intervention will be implemented over three years in approximately 90 acute care Ontario hospitals with high-volume EDs (those receiving >20,000 patient visits/annum). It will focus on organizational changes in three areas: more efficient processes (reforming/standardizing policies and practices); greater engagement of frontline staff in problem-solving; and supportive management systems. Modeled after three Ontario demonstration projects [16], the intervention is supported by a leadership and training program and organizational change experts in the form of coaching and training teams who facilitate the program in collaboration with local leaders and staff teams from participating hospitals. Change experts and hospital teams are tasked with improving processes from patient presentation in the ED to in-patient admission through to discharge by the integration of performance improvement pilot solutions across the ED and general medicine units.

In collaboration with senior decision makers at the Ontario MOH, a roll-out and evaluation strategy for the intervention was developed. The primary objective of the evaluation of the intervention is to determine whether the ED-PIP reduces total ED length-of-stay (ED-LOS). The secondary objectives are to determine the effects on time-to first physician contact and several measures of quality of care.

## Methods

We conducted a literature review to identify a list of possible minimization factors to guide the allocation of hospitals to the ED-PIP. Subsequently, a multi-stage modified-delphi expert panel process was performed that included candidate factor review, quantitative assessment, and a nominal group process in a final teleconference discussion.

### Literature review

To generate the list of candidate minimization factors, we reviewed databases from Management and Organizational Studies, PubMed/Medline and Ovid HealthSTAR using the search terms: organizational culture, healthcare/health system reform, transformation, intervention(s), context, evaluation, readiness for change, change management, implementation, process, and outcomes. We sought to identify articles and research papers specifically focused on organizational change and behaviour, change interventions, and research reports specific to healthcare and health services administration. One author (CL) examined all relevant references; candidate factors were considered regardless of any demonstrated empirical association to outcomes of the policy intervention under study.

The literature review [17-26] generated a preliminary list of potential factors associated with the success of organizational change interventions in healthcare settings. These were organized according to a published four-stage framework for healthcare professionals managing organizational change [20]. This framework builds on observational studies in change management literature and provides a model of change implementation in healthcare organizations, informed by the implementation of a major patient safety initiative at a large, multi-site, academic hospital in Toronto, Canada. Candidate factors were retained if they were relevant to the first three stages in the framework, which represent the most applicable domains of organizational capacity and readiness for change relevant to the implementation success of the ED-PIP. The last stage addresses long-term sustainability of change initiatives. Given the breath of indicators relevant to change stage two, we expanded this stage into two subcategories: organizational readiness for change; and situational analysis and redesign of organizational systems.

### Expert panel

We assembled an international multi-disciplinary panel of 21 experts consisting of hospital and ED administrators, physicians and nurse clinicians, health services and policy researchers, Ministry of Health senior leaders, organizational change researchers, and consultants with extensive experience in hospital change management interventions. Panelists represented health systems in Canada, the United Kingdom, and Australia. Diversity of experience from teaching and non-teaching hospitals was well represented among panelists. Consultants identified by two co-authors (RB, BG) were contacted and asked to nominate global experts who had experience facilitating organizational change management in health sectors abroad and were familiar with the proposed intervention concept.

### Modified-delphi and nominal group process

In a preliminary stage, panelists reviewed the list of factors generated from the literature review and were asked to suggest additional factors based on their knowledge of the literature and experience with health system improvement initiatives. A final list of candidate factors was generated and a two-round modified-delphi survey process followed. In round one, panelists rated candidate factors with respect to their expected correlation (high, low, or unsure) with the allocation strata for the intervention (hospital volume and geographic region). Previous research in Ontario suggests that variation in ED-LOS is based on ED volume and the geographic region of a given hospital [27]. Factors that were highly correlated with stratification variables were excluded because any confounding associated with them would be assumed to be dealt with through stratification. Panelists also rated the degree to which the factor would likely confound the effect of the ED-PIP on achieving improvements in ED-LOS and in-patient flow. Those rated as 'somewhat' and 'very' were coded as 'predictive – potential confounder', those rated as 'slightly' and 'not at all' were coded as 'not predictive – not a potential confounder'. Factors rated by greater than 70% of panelists as 'predictive – potential confounder' were retained for the second survey.

In order to obtain a broader perspective on potential confounders, we expanded the number of participants for the second survey [28,29]. In this phase, panelists rated each of the factors retained previously on a scale of one to nine, where one was 'completely disagree' and nine was 'completely agree' for the following three statements:

1. The factor measures a core component of a hospital's readiness to implement and facilitate an organizational change policy intervention aimed to improve ED-LOS and in-patient flow through to discharge.

2. The factor is highly predictive of the capacity for an organization to successfully implement the intervention and achieve improvements in patient flow.

3. The factor is evidence-based and linked to a hospital's ability to manage change activities related to the patient flow intervention.

A final score for each factor was derived by averaging the responses from the three questions noted above (a + b + c/3). Results were reviewed by panelists and discussed among the core group of panelists via teleconference guided by the nominal group technique. The highest ranking factor for each change stage domain was brought forward for discussion, definition, and specification of a measurement scale. The resulting minimization instrument was pilot tested using a web-based survey to Chief Executive Officers from six hospitals chosen to pilot the ED-PIP intervention. Hospitals were selected by the Ministry of Health. We categorized responses from one to nine as: lowest (one to three); moderately low (four, five); moderately high (six, seven); and highest (eight, nine). This study was approved by the Sunnybrook Health Sciences Centre Research Ethics Board (reference number 324-2007).

## Results

A total of 33 candidate minimization factors were generated from a literature review and initial consultation with panelists (See Additional file 1). Candidate factors related to the implementation of the ED-PIP and covered a broad spectrum of issues (see Appendix 1).

The first round questionnaire was circulated to the core group of panelists (n = 19); 11 (59%) panelists completed it. Twelve of the original 33 (36%) factors were retained for the second survey. The second round questionnaire was distributed to 21 panelists, (original 19, plus 2 international representatives) and 17 (80%) panelists completed it. Table 1 lists the second round questionnaire results for all 12 indicators emerging from the original 33. For each change stage, the top ranking factors across the domains were discussed; the factors with the highest average score in each domain were confirmed in the discussion as the consensus choice to include in the minimization algorithm. Panelist discussion via teleconference using the nominal group technique served to further clarify factor definition, appropriate wording, and response scale (one to nine) for the short survey instrument. The final four minimization factors are listed in Table 2.

**Table 1:** Factors relating to achievement of a patient flow improvement – organizational change policy intervention

	**Assessment Domains**			
	Organizational Readiness	Predictive of successful implementation	Capacity to manage change	Mean

**Change stage one: organizational goals & architecture**				

Please tell us to what extent your organizational leadership and/or organizational staff are concerned about ED-GIM (emergency department – general medicine) flow issues in your hospital:	7.7	6.7	5.4	**6.6**

ED-GIM flow issues in my hospital represent a critical challenge to our mission:	7.6	7.3	5.7	**6.6**

How high on your priority list would you place an initiative dealing with ED-GIM flow?	7.9	7.5	5.8	**7.1**

Is general internal medicine (GIM)/general medicine a core clinical priority for your hospital?	6.7	6	5.2	**6.0**

**Change stage 2a: organizational readiness for change**				

Please tell us your previous experience with organizational change initiatives: How many MAJOR organizational change initiatives have taken place or have been planned in the past year (2008/2009).	6.1	5.8	5.2	**5.7**

Thinking about your hospital, what is the significance of: Staff burn-out from past change initiatives, as a potential barrier to improvements in ED flow and efficiency?	6.5	6.6	5.5	**6.2**

Thinking about your hospital, what is the significance of: Physician resistance to change, as a potential barrier to improvements in ED flow and efficiency?	7.3	7.7	6.6	**7.2**

**Change stage 2b: situational analysis and redesign of organizational systems**				

Thinking about your hospital, what is the significance of: Current communication practices between physician leadership and front-line nursing management, as a potential barrier to improvements in ED flow and efficiency?	6.4	6.8	5.4	**6.2**

Thinking about your hospital, what is the significance of: Current lack of coordination between ER and internal medicine on bed management issues, as a potential barrier to improvements in ED flow and efficiency?	6.9	7.2	5.7	**6.6**

Thinking about your hospital, what is the significance of: Current lack of physician coverage in the ED, as a potential barrier to improvements in ED flow and efficiency?	6.5	6.3	5.5	**6.1**

**Change stage 3: capacity to build coalitions, broaden support and align systems**				

Considering previous change initiatives your hospital has undertaken, were you able to develop effective communication methods, systems and strategies within and between medical/clinical services and sub-specialists within your hospital?	6.3	6.5	5.9	**6.2**

Thinking about your hospital, what is the significance of: misalignment between physician incentives and goal of patient flow improvement, as a potential barrier to improvements in ED flow and efficiency?	6.8	7.4	6.5	**6.9**

**Table 2:** Minimization variables

**Change stage 1: organizational goals and architecture**
To what extent would an initiative aimed to optimize in-patient flow and reduce emergency department length of stay be considered as the foremost priority for your hospital's leadership in 2009–2010?
**Change stage 2a: organizational readiness for change**
How would you rate receptiveness to organizational change among physicians currently practicing at your hospital?

**Change stage 2b: situational analysis and redesign of organizational systems**
How would you rate the efficiency of bed management/coordination currently in practice between the emergency department and in-patient medical care units at your hospital?

**Change stage 3: capacity to build coalitions, broaden support and align systems**
State the degree to which physician incentives at your hospital are supportive of an organizational goal to optimize in-patient flow and reduce emergency department length of stay.

A total of six CEOs from a selected sample of ED-PIP hospitals received an invitation to complete the online survey and all (100%) completed it. The CEOs who scored each factor highest, moderately high, moderately low and lowest were as follows, Factor 1: 4,0,1,1; Factor 2: 1,3,2,0; Factor three: 0,5,1,0; and Factor four: 0,2,2,2.

## Discussion

Using a combined approach of evidence synthesis and a modified-delphi panel and nominal group process we identified four factors to be used in a minimization algorithm to guide the allocation of hospitals to the ED-PIP intervention. This structured panel process reduced 33 initial candidate factors to four, expressed as a simple four-item quantitative survey instrument. To our knowledge, this is the first published example of a minimization algorithm being used to allocate hospitals to a major health system policy intervention.

The intervention being developed to improve patient flow is complex, and complex interventions generally demonstrate modest gains in empirical study [30]. Evaluating such interventions requires careful balance of known and unknown confounders, because the effect of confounders may exceed the effect of the intervention, in either direction, to create a benefit that is either not real or hide a benefit that is real. This is an important advantage of randomized studies (and one which policymakers are generally not aware of), and pragmatic randomized trials of complex interventions can be designed so that they are no more difficult for policy makers to implement, and evaluative rigor is ensured. This can be especially important when the number of intervention units is small, say less than a hundred hospitals, rather than several hundred or several thousand patients as is more typical in patient-level intervention studies.

The disadvantages of randomized trials in the healthcare system include their cost, complexity, and the desire for rapid changes evidenced within political mandates (randomized controlled trials take considerable time). Due to these issues, decision makers often implement non-randomized observational designs (*e.g*., before-after) that are vulnerable to confounding and offer relative uncertainty with regard to understanding the true impact of transformative efforts to improve system performance, accountability, and quality of care to the consumer. Methods such as matching or stratifying by factors such as geography, hospital type, or volume are appropriate means to balance some confounders, but there is a limit to the number of strata one may use; minimization offers an alternative or complementary approach to ensure allocation is balanced with respect to important confounders of the ED-PIP intervention.

The minimization algorithm aims to ensure unbiased allocation of the intervention during its phased roll-out. Each factor has been defined in the form of a question with a nine-level response scale. Responses from volunteering hospitals will be assessed for variance and grouped into two levels (zero 'low' and one 'moderate/high') accordingly for evaluation in the minimization algorithm. The algorithm allocates the first hospital in presenting sequence of eligibility to receive the intervention in the first (year one) or later phases of implementation at random. The algorithm then allocates subsequent hospitals to each respective phase of the intervention minimizing differences across factor levels, such that, in each phase of implementation the sample is balanced with respect to hospitals with both low and moderate/high levels of each factor. In our pilot testing, we observed substantial variability between the six respondents on three of the four factors, suggesting that our minimization factors do discriminate and are suitable for use in the minimization algorithm to guide the allocation of the intervention to hospitals. All respondents rated factor three (effectiveness of bed-management) as 'moderately high'. It will therefore be important to monitor the variability in this factor when the survey is completed by CEOs from additional hospitals in Ontario as the ED-PIP is rolled out. Further pilot testing in additional hospitals is likely required before this tool can be widely recommended.

The organizational change management literature contains a large number of potential factors or mechanisms likely to represent either a barrier or facilitator to achieving change [17,19,20,23,31-39]. These are largely based on retrospective cross-sectional observation and evaluation of change interventions [40]. There are few longitudinal [41] studies or rigorous evaluations of these factors [42]. Gustafson and colleagues [39], however, offer a concise review of potential factors; and illustrate and test an 18-factor model devised to predict and explain the success or failure of a change process in healthcare settings. The model was derived from an expert panel process and literature review, but was neither evaluated with respect to objective outcomes nor designed to be used for intervention allocation purposes. Rather, the factors were compiled to guide managers initiating and managing a change initiative within a healthcare setting on actionable determinants of implementation success. The model is too complex for allocation using a minimization algorithm due to the number of factors and levels within each. Further, most factors are concerned with optimal intervention design and implementation rather than organizational culture or context factors likely to confound intervention success or failure. Our four factors are not designed as a comprehensive list of all potential factors affecting the success of a hospital level policy intervention, but rather as important hospital-specific factors likely to confound the success or failure of the intervention at all phases of implementation.  

Some study limitations are worth noting with respect to our process to define potential determinants to implementation success of the ED-PIP. While our literature review was comprehensive, it was confined to English peer-reviewed publications and may not have identified all possible previously cited factors. Our consultation with the panel of experts, however, did yield additional factors in the preliminary exercise. The minimization factors were developed with specific reference to the ED-PIP intervention; therefore, the four factors we identified may not necessarily be relevant for other hospital-level interventions. However, many of the obstacles to organizational change in healthcare settings potentially affecting success of a patient flow improvement initiative are likely common to other interventions as well. Indeed, our factors are similar to previously cited themes of obstacles to implementation success described in organizational change research within and beyond the health sector [18,19,22,26,31,37-39,43]. While our pilot results suggest reasonable variability across the four factors, we suggest caution to researchers who may wish to use these factors in other settings; piloting the instrument in a small number of centres prior to allocation based on these minimization factors is advisable.

Finally, the international membership of our panel made an in-person meeting prohibitively costly; however, regular electronic contact was maintained and timely feedback occurred. Biases may have resulted during the in-person/teleconference panel meeting from single panelists whose opinion may have been overly influential; however, the teleconference method may have mitigated this, and input was actively sought from all attendees.

## Conclusion

Change in all industries is difficult, perhaps in none more so than healthcare, where multiple stakeholders, sometimes conflicting missions and goals, professional independence of key staff, and difficulty accessing high-quality performance data present particular challenges [20]. Policies and interventions to improve hospital performance frequently require significant human and financial resource inputs, and rigorous evaluation is necessary both to evaluate their effectiveness and to better understand organizational factors contributing to success [44,45]. The evaluative strategy for the ED-PIP ensures that the intervention can be implemented in a way that is consistent with the needs of policy and health system decision makers, while at the same time offering a study design that provides for a rigorous evaluation of its effect on patient LOS in the ED.

## Competing interests

The authors declare that they have no competing interests.

## Authors' contributions

MS, AG, MZ, GA, TS, BG, BR, RB, DM and HA conceived of the study and design to systematically identify minimization factors, participated in the expert panel review process; and helped to draft the manuscript. CL carried out the literature review, coordinated and synthesized results from the panelist surveys; and drafted the manuscript. MS facilitated the teleconference. All authors read and approved the final manuscript.

## Appendix 1: main themes of candidate minimization factors

• Leadership/staff concern/prioritization of patient flow issues

• Historical experience with change initiatives (such as: total number in the past year, intensity of previous initiatives upon staff, number of planned initiatives for the upcoming year).

• Organizational infrastructure (such as: number of general internal medicine beds, effectiveness of bed management, information technology and decision support).

• Communication culture across professional groups.

• Capacities for participatory and collaborative engagement (such as: assessments of staff burn-out and staff capacity/resistance to lead, finance, or resource a change initiative).

• Importance of added values embedded in the intervention (such as: training opportunities, communication development strategies).

## Supplementary Material

Additional file 1 **Candidate factors by change stages**. Table lists 33 candidate factors by organizational change stages that the expert panel assessed across specified domains. Click here for file
