# Supplementary material for: Development of a minimization instrument for allocation of a hospital-level performance improvement intervention to reduce waiting times in Ontario emergency departments
Source: Implement Sci. 2009 Jun 8;4:32. doi: 10.1186/1748-5908-4-32 (PMC2706789; doi:10.1186/1748-5908-4-32)
Supplement: Additional file 1 — Candidate factors by change stages. Table lists 33 candidate factors by organizational change stages that the expert panel assessed across specified domains. [file 1748-5908-4-32-S1.pdf]

Appendix 1. Candidate factors by change stages

Change stage 1 — organizational goals and architecture

1. To what extent are your organizational leadership and/or organizational staff concerned about emergency department-general medicine (ED-GIM) flow issues in your hospital?
2. ED-GIM flow issues in my hospital represent a critical challenge to our mission.
3. How high on your organization's priority list would you place an initiative dealing with ED-GIM flow?
4. Is general internal medicine (GIM)/general medicine a core clinical priority for your hospital?
5. What proportion of beds in your hospital are dedicated general internal medicine (GIM)/general medicine beds?

Change stage 2a — organizational readiness for change

6. How many change initiatives has your hospital completed in the past four years?
7. How many major organizational change initiatives have taken place or have been planned in the past year?
8. In general, how would you rate your hospital's success with previous organizational change initiatives?
9. In general, how would you rate your staff's capacity to lead and participate in policy and management change initiatives?
10. Are you aware of any major institutional change initiatives or challenges that your hospital will face in the next year?
11. In the last year, has your hospital experienced any event that you would consider to be a crisis?
12. How long has your current CEO been in place?
13. How long has your hospital's physician in chief (PIC) been in place?
14. How long has your hospital's nurse in chief (NIC) or head of nursing been in place?
15. Are the current lack financial resources for performance improvement initiatives a potential barrier to improvements in ED flow and efficiency?
16. Is the current lack of GIM beds in your organization/facility a potential barrier to improvements in ed flow and efficiency?
17. Is difficulty recruiting/retaining nursing staff a potential barrier to improvements in ED flow and efficiency?
18. Is a current state of staff burn-out from past change initiatives a potential barrier to improvements in ED flow and efficiency?
19. Is physician receptiveness/resistance to change a potential barrier to improvements in ED flow and efficiency?
20. Does your hospital currently have a staff position or positions dedicated to leading and managing change initiatives?

Appendix 1. Candidate factors by change stages

Change stage 2b — situational analysis and redesign of organizational systems

21. Is a current lack of it/decision support infrastructure at your hospital a potential barrier to improvements in ED flow and efficiency?

22. Are current communication practices between physician leadership and front-line nursing management a potential barrier to achieving improvements in ED flow and efficiency?

23. Is there a current lack of coordination between ED and internal medicine on bed management issues at your organization?

24. Is there a current lack of physician coverage in the ED at your organization?

Change stage 3 — capacity to build coalitions, broaden support and align systems

25. In general, how would you rate your staff's receptiveness to support change initiatives?

26. Considering previous change initiatives your hospital has undertaken, how important do you think it is to include: training opportunities?

27. Considering previous change initiatives your hospital has undertaken, how important do you think it is to include: facilitating local ownership through consultation?

28. Considering previous change initiatives your hospital has undertaken, how important do you think it is to include: development of effective communication methods, systems, and strategies within and between medical/clinical services and sub-specialists within your hospital?

29. Is misalignment between physician incentives and goal of patient flow improvement a potential barrier to improvements in ED flow and efficiency?

30. Are existing communication practices between different professional groups in the organization/facility on the whole a potential barrier to improvements in ED flow and efficiency?

31. Is timely review of the emergency department consults to clinical services (diagnostic imaging and sub-specialist consultation) a potential barrier to improvements in ED flow and efficiency?

32. Does your hospital require the involvement of sub specialists prior to providing access to certain diagnostic imaging tests or reading/interpretation of tests for the emergency department?

33. Does access to clinical and/or medical services in your hospital have priority over the emergency department?
